# Hyperthyroidism increases the risk of osteoarthritis in individuals aged 60–80 years

**DOI:** 10.1038/s41598-024-64676-3

**Published:** 2024-06-17

**Authors:** Jinlong Zhao, Haodong Liang, Guihong Liang, Kunhao Hong, Weiyi Yang, Minghui Luo, Lingfeng Zeng, Jun Liu

**Affiliations:** 1 State Key Laboratory of Traditional Chinese Medicine Syndrome/The Second Clinical College of Guangzhou University of Chinese Medicine, No.111, Dade Road, Yuexiu District, Guangzhou, 510405 Guangdong China; 2 Guangdong Provincial Key Laboratory of Chinese Medicine for Prevention and Treatment of Refractory Chronic Diseases/The Second Affiliated Hospital of Guangzhou University of Chinese Medicine (Guangdong Provincial Hospital of Chinese Medicine), Guangzhou, 510120 China; 3 The Research Team On Bone and Joint Degeneration and Injury of Guangdong Provincial Academy of Chinese Medical Sciences, Guangzhou, 510120 China; 4 The Fifth Clinical College of Guangzhou, University of Chinese Medicine, No.12, Jichang Road, Baiyun District, Guangzhou, 510405 Guangdong China; 5 The Affiliated TCM Hospital of Guangzhou Medical University, Guangzhou, 510000 China; 6 Guangdong Second Traditional Chinese Medicine Hospital (Guangdong Province Engineering Technology Research Institute of Traditional Chinese Medicine), Guangzhou, 510095 China

**Keywords:** Hyperthyroidism, Osteoarthritis, Cross-sectional study, Mendelian randomization, NHANES, Medical research, Risk factors

## Abstract

To elucidate the currently unknown relationship between hyperthyroidism and osteoarthritis (OA). During 2007–2012, 7,433 participants (hyperthyroidism patients = 125; OA patients = 675) were included in the National Health and Nutrition Examination Survey database. We used a weighted multivariable-adjusted logistic regression analysis to assess the association between hyperthyroidism and OA. We also assessed the causality of that relationship using publicly available genome-wide association study data and three Mendelian randomization (MR) analysis methods. The heterogeneity test, pleiotropy test, and leave-one-out tests were used for sensitivity analysis. In this cross-sectional study, after adjusting for potential confounding factors, we found that hyperthyroidism significantly (*P* = 0.018) increased the risk of OA (odds ratio [OR] = 2.23, 95% confidence interval [CI] = 1.2–4.17). Age-stratified analysis revealed that hyperthyroidism was associated with a greater risk of OA in the 60–80-year-old age group (OR = 2.86, 95% CI = 1.46–5.59, *P* = 0.002), with no significant association in the 18–59-year-old age group (all *P* > 0.05). The results of the inverse-variance weighting (IVW) analysis showed that hyperthyroidism increased the risk of OA (OR = 1.23, 95% CI = 1.04–1.46; *P* = 0.017). The weighted median estimator (WME) and MR-Egger method also confirmed this causal association (OR = 1.27 and OR = 1.32, respectively). The sensitivity analysis results confirmed the reliability of this conclusion. In addition, IVW-based reverse-MR analysis revealed that OA did not increase the risk of hyperthyroidism (OR = 1.02, 95% CI = 0.97–1.08;* P* = 0.449). Hyperthyroidism is associated with an increased risk of OA, but the underlying pathological mechanism still needs to be clarified in future research.

## Introduction

Osteoarthritis (OA) is a degenerative bone and joint disease caused by articular cartilage injury, hyperosteogeny, subchondral osteosclerosis, and pathological changes in the synovium and/or articular cavity^1,2^ and is mainly characterized by joint pain and dysfunction. The pathogenesis of OA is relatively complex and is related to factors such as inflammation, loss of joint fluid, cytokine levels, and metabolic abnormalities^3,4^. OA typically occurs among middle-aged and elderly people and has a high incidence, disability rate, and mortality rate, imposing a serious economic burden on patients' families and society^2,5^. A study based on the global burden of disease data showed that^6^ compared with that for lower back and neck pain, the age-standardized incidence rate (ASIR) for OA increased by 0.32% (95% CI 0.28–0.36) annually worldwide or approximately 9% over 28 years (from 1990 to 2017). The recognized causes of OA include obesity, genetic factors, ageing, inflammation, and trauma^3,4,7^, and joint cartilage degeneration, caused by the combined effects of these factors, is the core link in the pathogenesis of OA. In addition, some disease-related factors, such as fatigue and infection events, have also been found to be associated with OA^8–10^. Heijman et al. reported that OA patients experience a stable daily level of fatigue^9^, which means that OA patients have a worse quality of life than healthy people. Patients with end-stage OA may inevitably need surgical treatment^10^, and possible infection events may not only increase the health and economic burden but also seriously reduce quality of life. Undoubtedly, early identification of controllable factors that increase the risk of OA and management of these factors have important clinical and public health value in reducing the incidence of OA or delaying its progression.

Thyroid dysfunction is believed to be associated with OA^11,12^, but strong evidence supporting this potential correlation is lacking. Krieger et al.'s research showed that thyroid-stimulating hormone (TSH) and other thyroid hormones (THs) can induce the occurrence of OA^13^. Numerous studies have shown that TSH can act on target organs outside the thyroid gland (such as articular cartilage)^14–16^ and is involved in the occurrence and development of various diseases. Hyperthyroidism is a common disease of the endocrine system caused by an increase in the synthesis or secretion of THs^17^. A clinical study involving 109 patients with thyroid dysfunction revealed a significant association between hyperthyroidism and knee joint pain; the included patients had abnormal muscle–bone ultrasound findings^12^. However, there is still a lack of evidence supporting the association between hyperthyroidism and OA. To fill this gap, we utilized data from the National Health and Nutrition Examination Survey (NHANES) database to investigate the relationship between hyperthyroidism and OA, which was our primary research goal. For our second research goal, we used a two-sample Mendelian randomization (MR) method to explore whether there was a causal relationship between hyperthyroidism and OA. Our hypothesis was that there is a positive causal relationship between hyperthyroidism and OA and that there is no reverse causal relationship. The conclusions of this study can provide a reliable basis for preventing or delaying the progression of OA in hyperthyroidism patients.

## Materials and methods

### Cross-sectional study

#### Data sources and study participants

**Figure 1:**
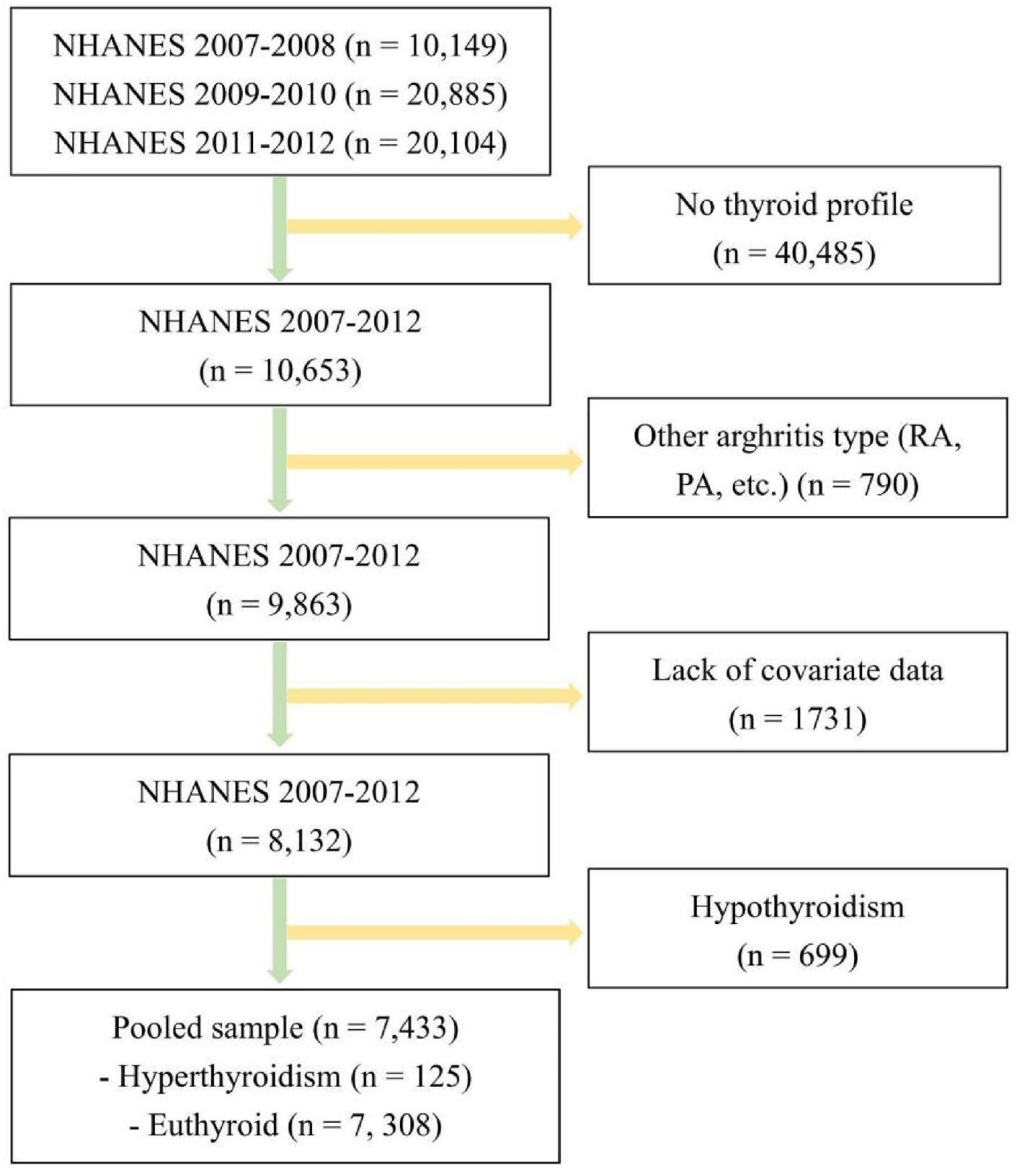
Flow chart of study participants.

We used NHANES data from 2007 to 2008, 2009 to 2010, and 2011 to 2012; laboratory data on thyroid function were reported during these three survey cycles. The NHANES is a cross-sectional survey conducted by the Centers for Disease Control and Prevention (CDC) in the United States^18^. All participants signed an informed consent form^19^, and the use of NHANES data was approved by the Ethics Review Committee of the National Center for Health Statistics in the United States^19^. Thus, no external ethical approval or informed consent was needed. For the 2007–2012 survey period, a total of 10,600 participants had complete thyroid function measurement data. To accurately study the relationship between hyperthyroidism and OA, we excluded participants with rheumatoid arthritis, other types of arthritis, psoriatic arthritis, or hypothyroidism. In addition, all participants lacking OA status and thyroid function information were not included for further analysis, and 7433 participants were ultimately included. The inclusion and exclusion process for the research participants is shown in Fig. 1.

#### Determination of OA outcomes

The diagnosis of OA was mainly determined from the questionnaire survey results. In the NHANES questionnaire, the participants were asked "Has a doctor or other health professional ever told you that you have arthritis?". If a participant answered “yes”, the question "Which type of arthritis do you have?" was subsequently asked. Participants who self-reported having osteoarthritis were considered OA patients for the purpose of the present study. Previous studies have confirmed the effectiveness of self-reported OA diagnoses^20,21^.

#### Measurement of thyroid function

The diagnosis of hyperthyroidism was based on the participants’ reported drug use and TSH test results, and this evaluation method was similar to that used by Kakigi et al. and Airaksinen et al.^22,23^. We collected TSH data measured by the NHANES from 2007 to 2012, as well as data on drug usage. To evaluate thyroid function, blood samples were collected from all participants at the NHANES Mobile Examination Center (MEC) and transported to the collaborating laboratory service centre in Ottumwa, Iowa^24^. TSH measurements were quantitatively analysed using the third-generation dual-site immune enzyme high-sensitivity human TSH method^24^. According to the reagent manufacturer guidelines, the normal TSH range is 0.34–5.6 mIU/mL^25^. Participants who had not received TH replacement treatment or antithyroid drug treatment and whose TSH value was 0.34–5.6 mIU/mL were classified as having normal thyroid function^26,27^. Participants who self-reported current levothyroxine use, had a desiccated thyroid (regardless of their TSH level) or whose TSH level was greater than 5.6 mIU/mL and who did not take antithyroid drugs were classified as having hypothyroidism^26,27^. Participants who reported current methimazole or propylthiouracil use (regardless of their TSH level) or who had a TSH level less than 0.34 mIU/mL were classified as having hyperthyroidism^26,27^.

#### Assessment of covariates

The covariates included in this cross-sectional study were mainly sociodemographic factors and included age, sex, race, body mass index (BMI), smoking status, and alcohol consumption. We divided the patients into three groups based on BMI values: < 25.0, 25.0–29.9, and ≥ 30 kg/m^2^. According to Rattan et al.'s classification criteria^28^, we classified alcohol consumption levels into never, former, low, moderate, and heavy. In addition, we included six diseases, diabetes^29^, chronic kidney disease (CKD)^30^, hypertension^31^, hyperlipidaemia^32^, chronic obstructive pulmonary disease (COPD)^33^, and coronary heart disease (CHD)^34^, as covariates. The diagnoses for these six diseases were based on clear diagnostic criteria^29–34^ and matched with measurement indicators in the NHANES database; the participants included in this study voluntarily reported taking disease-related drugs, such as hypoglycaemic drugs and antihypertensive drugs, or reported having been diagnosed by a doctor. In addition, to further explore the association between hyperthyroidism status and the risk of developing OA in different age groups, subgroup analysis was also performed in this study.

### MR study

#### Assumptions and data sources for the MR analysis

**Figure 2:**
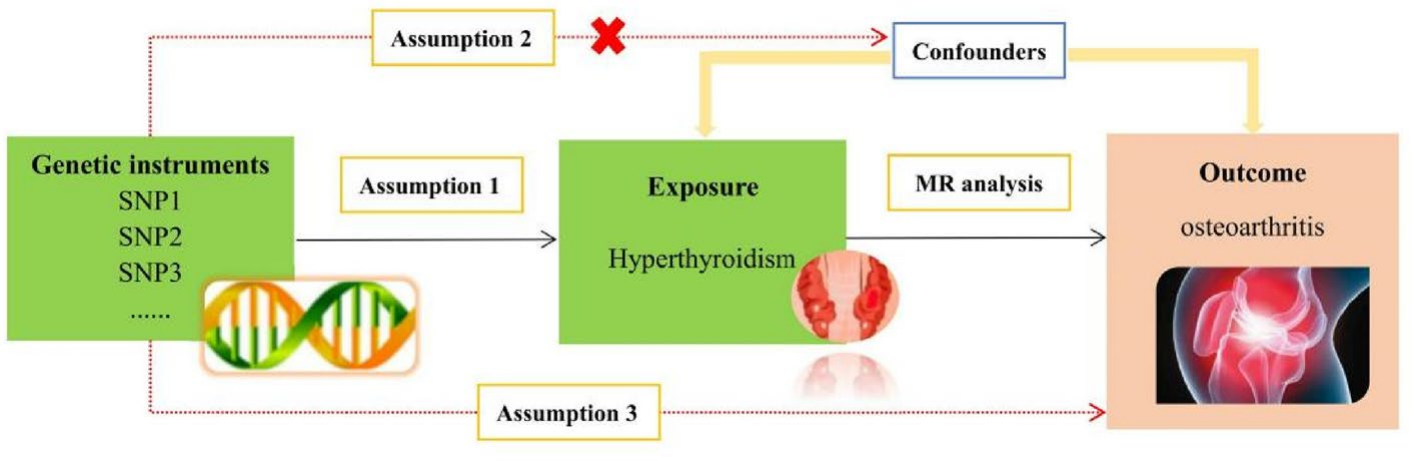
The schematic diagram of two- sample MR analysis.

MR analysis is considered to meet the three core assumptions of relevance, independence, and exclusivity, which are the foundation of this type of analysis. In the MR analysis in this study, the three specific assumptions are summarized as follows: (1) the instrumental variables (IVs) had to be strongly related to the exposure factors (hyperthyroidism); (2) the IVs could not be associated with any confounding factors associated with the "exposure-outcome"; and (3) the IVs could only affect the outcome variable (OA) through exposure factors (hyperthyroidism) and could not be affected by other factors. The hypothesis for the MR analysis in this study is shown in Fig. 2. The genome-wide association study (GWAS) target population consisted entirely of Europeans, and both males and females were included in this study. The data source was the Integrative Epidemiology Unit (IEU) analysis of UK Biobank phenotypes (https://gwas.mrcieu.ac.uk/). The GWAS included data from 337,159 hyperthyroidism patients, with 2547 included in the case group and 334,612 included in the control group (ID: ukb-a-76). A total of 10,894,596 single nucleotide polymorphisms (SNPs) were detected. The sample size in the OA GWAS data was 462,933 participants, including 38,472 OA patients and 424,461 non-OA patients (ID: ukb-b-14486), with a total of 985,1867 SNPs. The specific information for the data sources is provided in Supplementary Material 1.

#### Genetic variants associated with hyperthyroidism

The selection of IVs satisfied the first assumption of the MR hypothesis in this study; that is, IVs (SNPs) had to have a strong correlation with hyperthyroidism. With *P* < 5 × 10^−8^ as the screening criterion, SNPs that were strongly correlated with hyperthyroidism were screened from the GWAS data. On this basis, we set the parameters (distance > 10 MB and *r*^2^ < 0.001) to run the data filtering program to conduct linkage disequilibrium pruning of the SNPs. If there was no strong correlation between the genetic variation and the exposure factors or if the genetic variation could only explain a small part of the phenotypic variation, the genetic variation was referred to as weak^35^. We evaluated whether the IVs selected were weak by calculating *F* statistics^35^.

Second, to ensure that the second MR hypothesis was met, we queried all the IVs that met the assumption of the first hypothesis in the PhenoScanner database (http://www.phenoscanner.medschl.cam.ac.uk/) to ensure that these IVs were not associated with known confounding factors (BMI, age, oestrogen reduction) (Supplementary Material 2).

Finally, we extracted the SNPs selected in the steps described above from the GWAS data of the outcome variable (OA). In addition, SNPs related to OA were excluded (*P* < 5 × 10^−8^).

### Statistical analysis

#### Cross-sectional analysis

The data analysis strategy in this section was conducted in accordance with the CDC's statistical guidelines^36^. All data analyses were conducted using R software (version 4.2.2, http://www.R-project.org, The R Foundation) and EmpowerStats software (version 4.1, www.empowerstats.com, X&Y solutions, Inc. Boston, MA, USA). All tests were bilateral and had a significance level of α = 0.05. Categorical variables are expressed as frequencies and weighted percentages. We used a binary logistic regression model to evaluate the relationship between hyperthyroidism and OA. To test the stability of the relationship between hyperthyroidism and OA, we constructed three regression models to gradually adjust for confounding factors: Model 1 was not adjusted for covariates; Model 2 was adjusted for age, sex, BMI, and race; and Model 3 expanded on Model 2 and was further adjusted for smoking status, alcohol consumption, diabetes status, CKD status, hypertension status, hyperlipidaemia status, COPD status, and CHD status.

#### MR analysis

MR analysis was performed using the TwoSampleMR package in R software (version 4.2.2, http://www.R-project.org, The R Foundation). We used inverse variance weighting (IVW) random effects, the weighted median estimator (WME) and MR‒Egger for MR analysis and evaluated the potential causal relationship between hyperthyroidism and OA with odds ratios (ORs) and 95% confidence intervals (CIs). The principle of IVW is to calculate the weighted average of all IVs' effect sizes on the premise of ensuring that all IVs are valid and not considering intercept terms in regression analysis^37^, which is considered the main statistical method of MR analysis.

To evaluate whether OA had a causal effect on hyperthyroidism, we conducted reverse MR analysis with OA as the exposure and hyperthyroidism as the outcome. The process for selecting IVs and for MR analysis is described above. The final IV information for the reverse MR analysis can be found in Supplementary Material 3.

To further test the validity and robustness of the statistical results, we conducted sensitivity analyses. In this MR analysis, three methods, namely, the heterogeneity test, pleiotropy test, and leave-one-out (LOO) sensitivity test, were used for sensitivity analyses^38,39^.

### Ethics approval and consent to participate

The protocols of NHANES were approved by the institutional review board of the National Center for Health Statistics, CDC (https://www.cdc.gov/nchs/nhanes/irba98.htm). NHANES has obtained written informed consent from all participants.

## Results

### Cross-sectional study

#### Baseline characteristics of the study participants

A total of 7,433 participants (18–80 years old) were included in this study, with 675 OA patients identified from personal interview data, including their self-reported health status; the remaining 6758 participants were all non-OA patients. In terms of thyroid function, a total of 125 participants (1.4%) were identified as having hyperthyroidism, while the other 7308 participants (98.6%) had normal thyroid function. The weighted percentages of hyperthyroidism incidence in OA and non-OA participants were 3.0% and 1.3%, respectively, and the difference between the two groups was statistically significant (*P* = 0.008). All covariates included in this cross-sectional study showed statistically significant differences between OA patients and non-OA patients (*P* < 0.05). The basic characteristics of the included participants based on OA status are shown in Table 1.

**Table 1:** Baseline characteristics of the included participants.

Variables	Total, N (weighted %)	Non-OA (n = 6758)	OA (n = 675)	*P*
		N (weighted %)	N (weighted %)	
Age, years				< 0.001
18–34	2113 (30.7)	2089 (33.3)	24 (3.6)	
35–49	1999 (31.1)	1914 (32.6)	85 (15.5)	
50–59	1109 (17.8)	999 (17.3)	110 (24.0)	
60–80	2212 (20.4)	1756 (16.8)	456 (56.9)	
Gender				< 0.001
Male	3866 (51.6)	3596 (52.5)	270 (41.9)	
Female	3567 (48.4)	3162 (47.5)	405 (58.1)	
Race/ethnicity				< 0.001
Non-Hispanic White	3235 (67.3)	2804 (65.8)	431 (83.1)	
Non-Hispanic Black	1494 (10.8)	1395 (11.2)	99 (6.2)	
American Mexican	1268 (8.9)	1220 (9.5)	48 (2.6)	
Others 4	1436 (13.0)	1339 (13.5)	97 (8.0)	
BMI (kg/m^2^)				0.0004
< 25	2269 (33.0)	2113 (33.8)	156 (25.5)	
25.0–29.9	2535 (34.2)	2317 (34.4)	218 (32.8)	
≥ 30	2532 (31.7)	2244 (30.8)	288 (40.6)	
Missing	97 (1.0)	84 (1.0)	13 (1.2)	
Smoking status				< 0.001
Never	4091 (54.8)	3763 (55.5)	328 (47.4)	
Current	1607 (21.8)	1501 (22.6)	106 (14.1)	
Former	1735 (23.4)	1494 (21.9)	241 (38.5)	
Alcohol consumption				< 0.001
Never	1613 (18.2)	1472 (18.1)	141 (18.9)	
Former	1218 (14.2)	1055 (13.5)	163 (21.5)	
Low to moderate	3071 (45.9)	2763 (45.6)	308 (48.8)	
Heavy	1531 (21.8)	1468 (22.9)	63 (10.8)	
Diabetes				< 0.001
No	6176 (88.1)	5664 (88.8)	512 (80.2)	
Yes	1257 (11.9)	1094 (11.2)	163 (19.8)	
CKD				< 0.001
No	6214 (88.4)	5720 (89.4)	494 (78.2)	
Yes	1219 (11.6)	1038 (10.6)	181 (21.8)	
Hypertension				< 0.001
No	4634 (67.6)	4390 (70.1)	244 (41.3)	
Yes	2799 (32.4)	2368 (29.9)	431 (58.7)	
Hyperlipidemia				< 0.001
No	2062 (28.3)	1932 (29.2)	130 (19.1)	
Yes	5371 (71.7)	4826 (70.8)	545 (80.9)	
COPD				< 0.001
No	7003 (94.1)	6403 (94.6)	600 (88.6)	
Yes	430 (5.9)	355 (5.4)	75 (11.4)	
CHD				< 0.001
No	7192 (97.4)	6564 (97.7)	628 (94.2)	
Yes	241 (2.6)	194 (2.3)	47 (5.8)	
Hyperthyroidism				0.008
No (euthyroid)	7308 (98.6)	6651 (98.7)	657 (97.0)	
Yes	125 (1.4)	107 (1.3)	18 (3.0)	

For categorical variables: N (survey-weighted percentage), *P*-value was by survey-weighted Chi-square test.

*N* number of observed, *BMI* body mass index, *CKD* chronic kidney disease, *COPD* chronic obstructive pulmonary disease, *CHD* coronary heart disease.

#### Association between hyperthyroidism and OA

We constructed three logistic regression models to explore the independent impact of hyperthyroidism on OA (Table 2). The unadjusted model (Model 1) results showed that hyperthyroidism significantly increased the risk of OA (OR = 2.4, 95% CI 1.23–4.71; *P* = 0.014). The results of the microadjustment model (Model 2) and the model adjusted for all covariates (Model 3) showed that hyperthyroidism increased the risk of OA, and the corresponding effect sizes (and 95% CIs) were OR = 2.11 (95% CI 1.15–3.87, *P* = 0.02) and OR = 2.23 (95% CI 1.2–4.17, *P* = 0.018), respectively. The results of the logistic regression analysis indicated a positive correlation between hyperthyroidism and the risk of OA.

**Table 2:** Association between hyperthyroidism and OA, NHANES 2007–2012.

Exposure	Model 1	*P*	Model 2	*P*	Model 3	*P*
	OR (95% CI)		OR (95% CI)		OR (95% CI)	
Hyperthyroidism (individuals aged 18–80)						
No (euthyroid)	Reference	0.014	Reference	0.02	Reference	0.018
Yes	2.4 (1.23, 4.71)		2.11 (1.15, 3.87)		2.23 (1.2, 4.17)	
Hyperthyroidism (subgroup: individuals aged 18–34 years)						
No (euthyroid)	Reference	0.989	Reference	0.989	Reference	0.992
Yes	0 (0, inf)		0 (0, inf)		0 (0, inf)	
Hyperthyroidism (subgroup: individuals aged 35–49 years)						
No (euthyroid)	Reference	0.858	Reference	0.987	Reference	0.783
Yes	0.83 (0.11, 6.2)		1.02 (0.13, 7.77)		0.75 (0.09, 5.92)	
Hyperthyroidism (subgroup: individuals aged 50–59 years)						
No (euthyroid)	Reference	0.674	Reference	0.621	Reference	0.452
Yes	0.65 (0.08, 4.96)		0.59 (0.07, 4.75)		0.44 (0.05, 3.69)	
Hyperthyroidism (subgroup: individuals aged 60–80 years)						
No (euthyroid)	Reference	0.004	Reference	0.004	Reference	0.002
Yes	2.52 (1.33, 4.76)		2.63 (1.36, 5.1)		2.86 (1.46, 5.59)	

inf: infinity; model 1: no covariates were adjusted; model 2: age (excluding subgroups), gender, BMI, and race/ethnicity were adjusted; model 3: age (excluding subgroups), gender, BMI, race/ethnicity, smoking status, alcohol consumption, diabetes, CKD, hypertension, hyperlipidemia, COPD, and CHD were adjusted. Note: Among the surveyed population aged 18–34 years, the number of individuals with hyperthyroidism in the OA group was 0.

Considering the close relationship between the OA status and age of the surveyed population, we analysed the association between hyperthyroidism status and OA risk in different age groups (18–34, 35–49, 50–59, and 60–80 years). The statistical results indicated that there was no significant correlation between hyperthyroidism status and the risk of OA in the populations aged 18–34 (*P* = 0.992), 35–49 (*P* = 0.783), or 50–59 (*P* = 0.452) years. In the population aged 60–80 years, hyperthyroidism was positively correlated with a greater risk of OA (OR = 2.86, 95% CI 1.46–5.59, *P* = 0.002). All these statistical results can be found in Table 2.

### MR study

#### Bidirectional MR analyses of hyperthyroidism and OA

**Figure 3:**
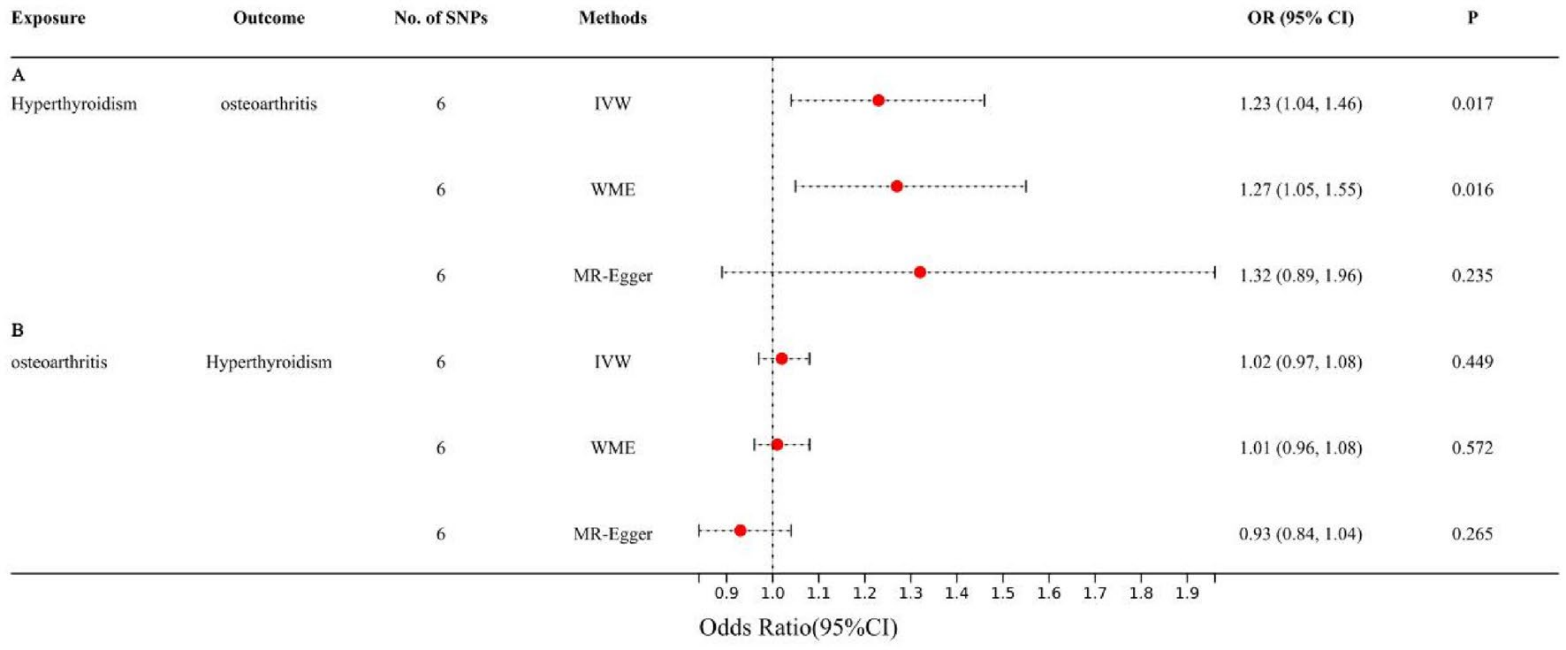
The forest plot for estimates of causal effects between hyperthyroidism and OA. (**A**) Taking hyperthyroidism as exposure; (**B**) taking OA as exposure.

To meet the three major assumptions of this MR study, a total of 6 SNPs were used for MR analysis (Table 3). MR analysis based on the IVW method revealed a causal relationship between hyperthyroidism and OA, indicating that hyperthyroidism increases the risk of OA (OR = 1.23, 95% CI 1.04–1.46; *P* = 0.017) (Fig. 3A). In addition, MR analysis based on the WME and MR-Egger methods also revealed that hyperthyroidism increases the risk of OA, and the statistical results obtained with these two methods were similar to those obtained with the IVW method (OR = 1.27 and OR = 1.32, respectively).

**Table 3:** 6 SNPs associated with hyperthyroidism.

SNP	Chr	Position	Effect allele	Other allele	EAF	Beta	Se	*P* for hyperthyroidism	*P* for OA	*F* statistic
rs1794279	6	32667595	T	G	0.1295	0.0071	0.0003	3.04E-111	0.03	503
rs2160215	14	81461472	C	T	0.3764	0.0025	0.0002	8.90E-31	0.36	133
rs28360997	6	31188654	A	G	0.1488	-0.0017	0.0003	6.01E-09	0.14	34
rs28383364	6	32606912	G	A	0.1965	-0.0033	0.0003	2.11E-35	0.3	154
rs3087243	2	204738919	A	G	0.4516	-0.0019	0.0002	3.80E-21	0.79	89
rs6679677	1	114303808	A	C	0.10257	0.0026	0.0003	4.42E-14	0.2	57

*Chr* chromosome, *EAF* effect allele frequency, *OA*: osteoarthritis.

To confirm whether there is a reverse causal relationship between hyperthyroidism and OA, we conducted reverse MR analysis (Fig. 3B). The IVW results showed that OA did not increase the risk of hyperthyroidism (OR = 1.02, 95% CI 0.97–1.08, *P* = 0.449), and this conclusion was also confirmed by the statistical results obtained with the WME (OR = 1.02, 95% CI 0.96–1.08, *P* = 0.572) and MR‒Egger methods (OR = 0.93, 95% CI 0.84–1.04, *P* = 0.265).

#### Sensitivity analysis

**Figure 4:**
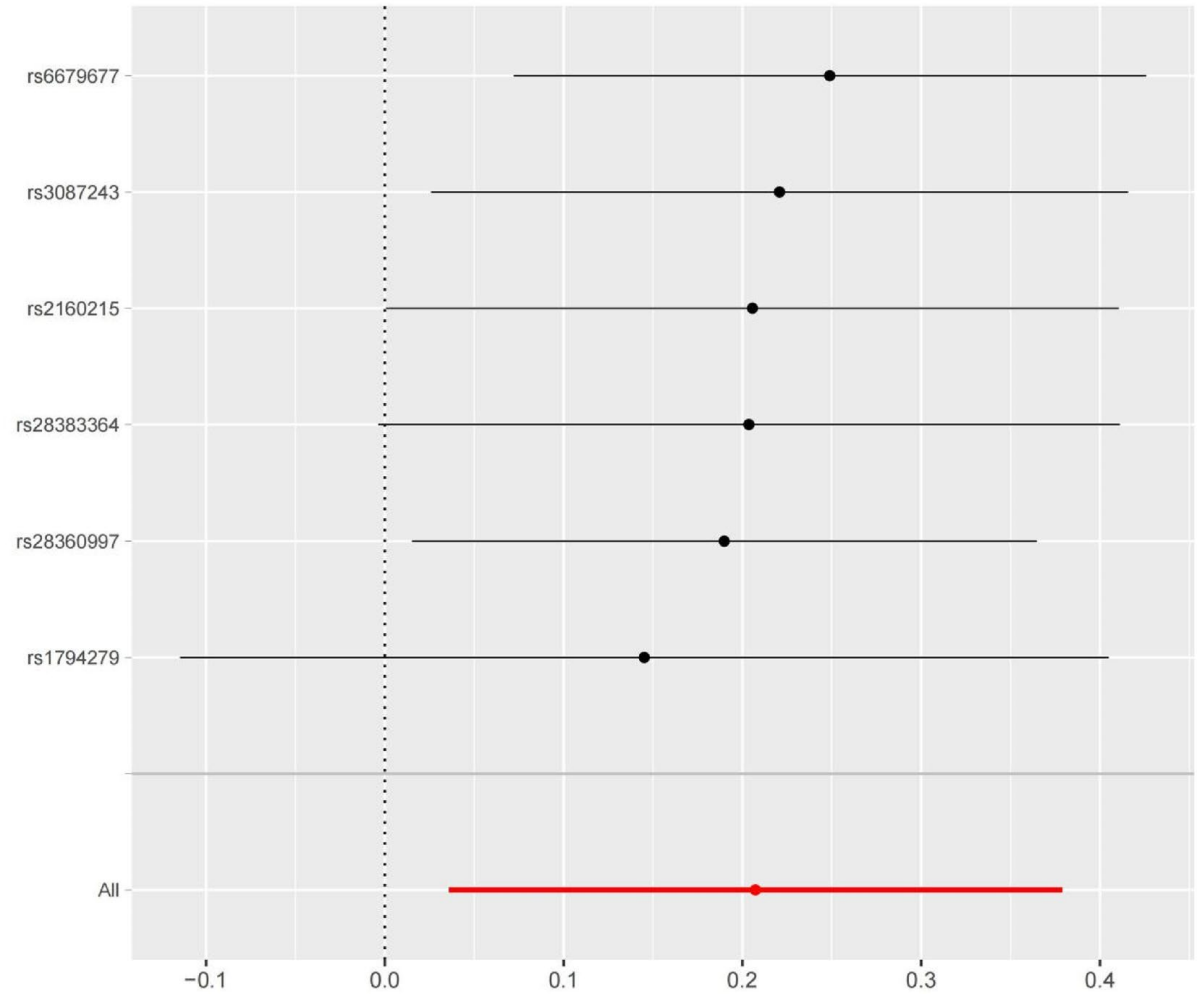
The forest map based on the analysis result of LOO.

The heterogeneity test results showed that the *Q* and *P* for the IVW and MR-Egger *Q* values were 4.91 (0.426) and 4.71 (0.318), respectively, indicating that heterogeneity did not exist. We used the MR-Egger regression intercept to verify the existence of pleiotropy in this study. The statistical results showed that the Egger intercept value was − 0.0002 (close to 0), and *P* = 0.699, indicating that there was no horizontal pleiotropy in this study. The LOO results showed that the causal relationship between hyperthyroidism and an increased risk of OA did not change after systematically removing a single SNP and repeating IVW-based MR analysis (Fig. 4).

## Discussion

There is a lack of strong research reporting on the association between hyperthyroidism and OA, and our study fills this gap. We explored the relationship between hyperthyroidism and OA based on cross-sectional NHANES and GWAS data and aimed to verify the potential causal relationship between hyperthyroidism and OA to provide convincing evidence. The results of this cross-sectional study indicate a positive linear relationship between hyperthyroidism and OA, indicating that hyperthyroidism increases the risk of OA in the population aged 60–80 years. In addition, two-sample MR analysis revealed that hyperthyroidism increases the risk of OA via a significant causal relationship, and this conclusion was not affected by a reverse causal relationship; that is, OA does not increase the risk of hyperthyroidism. The findings of this study will provide strong guidance for the prevention and treatment of OA, which means that identifying relevant targets for hyperthyroidism treatment may reveal new ideas for the pharmaceutical treatment of OA. In addition, the conclusions of this study also provide a basis for the public health system to strengthen the monitoring of OA occurrence in hyperthyroidism patients.

Cartilage injury, subchondral bone destruction, and chondrocyte hypertrophy are significant pathological features of OA^40,41^ and are also the main pathological factors for the occurrence and progression of OA. Rim et al. showed that the TSH receptor is highly expressed in chondrocytes^42^, but the role and mechanism of TSH in chondrocyte differentiation have not yet been revealed. One study showed that osteoporosis can affect overall bone mass^43^, and abnormalities in the microstructure of subchondral bone tissue may lead to uneven stress on articular cartilage, which may lead to secondary cartilage damage and osteophyte hyperplasia, thereby promoting the occurrence and progression of OA. Tokgoz et al. reported that greater bone mineral density (BMD) can delay the progression of knee osteoarthritis (KOA)^44^. The impact of thyroid-related hormone levels on bone tissue has long been studied by scholars^45,46^, and among these impacts, TH and TSH have positive regulatory and maintenance effects on bone development^47,48^. High TH levels in hyperthyroidism patients lead to an increase in the bone turnover rate and an imbalance in the proportion of bone resorption and bone formation processes^49^. Moreover, the inhibition of TSH can also affect the bone regulatory effect of TSH^49,50^, which can have a negative impact on the bone density of hyperthyroidism patients. In this context, hyperthyroidism patients have a greater risk of osteoporosis, which may be associated with the destruction of the subchondral bone microstructure; this may be a potential mechanism by which hyperthyroidism increases the risk of OA. Chen et al.'s cross-sectional study showed that conventional indicators of thyroid function (such as TSH and TH levels) cannot be independently used as predictors of the risk of OA^47^, but thyroid hormone indices, such as the thyroxine resistance index (TT4RI), thyroid feedback quantitative-based index (TFQI), and free triiodothyronine/free thyroxine (FT3/FT4) ratio, should be used. The findings of this study indicated that the FT3/FT4 ratio, TT4RI, and TFQI are closely related to the development of OA^47^, and the authors believe that there is a correlation between the thyroid system and chondrogenic differentiation, but that study does not provide information about the underlying mechanism involved. Although our study revealed a causal relationship between hyperthyroidism and OA, the pathological mechanism underlying the relationship between hyperthyroidism and OA is still unclear. Considering that hyperthyroidism is the cause of OA, future research needs to further clarify the possible pathogenic mechanisms, which may be a breakthrough point in discovering therapeutic targets for OA.

This study has the following limitations. First, due to the lack of thyroid function measurement data for some participants in the three survey cycles, we had to exclude these participants based on the scope of this study. Due to the absence of these samples, there may be bias in the conclusions of the cross-sectional analysis. Because the target population of this study included both males and females, menopause in females was not considered a covariate, and further exploration of the effect of menopause on hyperthyroidism and OA risk in the female population is needed in future research. Second, importantly, the target population of the cross-sectional and MR analyses was individuals living in the United States and Europe, which may make the conclusions of the study unsuitable for extrapolation to populations from other countries or continents. Thus, future research needs to include correlation and genetic-level causal analysis within the same sample population, which will provide additional scientific evidence for this topic. Finally, the MR study used aggregated GWAS data and lacked individual data, which made it impossible for us to conduct hierarchical analysis and make comparisons with NHANES population data. Therefore, future MR studies should include further analysis of the risk of hyperthyroidism and OA in the population aged 60–80 years.

## Conclusions

In conclusion, our cross-sectional and MR analyses showed that hyperthyroidism status increased the risk of OA, and this conclusion was reliable. The findings of this study will be beneficial for promoting the development of drug targets for OA and strengthening the monitoring of OA risk in hyperthyroidism patients. The pathological mechanism by which hyperthyroidism increases the risk of OA still needs to be clarified in future research.

### Supplementary Information

Supplementary Tables.

## Data Availability

The datasets generated and analyzed in the cross-sectional study are available at NHANES website: https://www.cdc.gov/nchs/nhanes/index.htm.

## Abbreviations

OA: Osteoarthritis
NHANES: National health and nutrition examination survey
GWAS: Genome wide association study
MR: Mendelian randomization
LOO: Leave-one-out sensitivity test
IVW: Inverse-variance weighting
WME: Weighted median estimator
ASIR: Age standardized incidence rate
TSH: Thyroid-stimulating hormone
TH: Thyroid hormones
CDC: Centers for disease control and prevention
MEC: Mobile examination center
OR: Odds ratio
CI: Confidence interval
SNPs: Single nucleotide polymorphisms
T3: Triiodothyronine
T4: Thyroxine
BMD: Bone mineral density
BMI: Body mass index
CKD: Chronic kidney disease
COPD: Chronic obstructive pulmonary disease
CHD: Coronary heart disease
IVs: Instrumental variables
