# Supplementary material for: Hyperthyroidism increases the risk of osteoarthritis in individuals aged 60–80 years
Source: Sci Rep. 2024 Jun 17;14:13924. doi: 10.1038/s41598-024-64676-3 (PMC11183066; doi:10.1038/s41598-024-64676-3)
Supplement: Supplementary file 1 — Supplementary Tables. [file 41598_2024_64676_MOESM1_ESM.docx]

**Supplementary Material 1 Summary of the GWAS included in this study.**

| Variable | ID | Sample size | Number of SNPs | Population | Sex | Year | Web source |
| --- | --- | --- | --- | --- | --- | --- | --- |
| osteoarthritis | ukb-b-14486 | 462,933 | 9,851,867 | European | Males and Females | 2018 | https://gwas.mrcieu.ac.uk/datasets/  ukb-b-14486/ |
| Hyperthyroidism | ukb-a-76 | 337,159 | 10,894,596 | European | Males and Females | 2017 | https://gwas.mrcieu.ac.uk/datasets/  ukb-a-76/ |

**Supplementary Material 2 Related phenotypes of 6 instrumental variables.**

| **snp** | **a1** | **a2** | **trait** | **pmid** | **year** | **beta** | **se** | **p** | **n** |
| --- | --- | --- | --- | --- | --- | --- | --- | --- | --- |
| rs1794279 | T | G | Basophil count | 27863252 | 2016 | -0.03968 | 0.005203 | 2.41E-14 | 173480 |
| rs1794279 | T | G | Eosinophil count | 27863252 | 2016 | -0.06924 | 0.005304 | 6.16E-39 | 173480 |
| rs1794279 | T | G | Eosinophil percentage of granulocytes | 27863252 | 2016 | -0.03788 | 0.005321 | 1.08E-12 | 173480 |
| rs1794279 | T | G | Eosinophil percentage of white cells | 27863252 | 2016 | -0.0386 | 0.005298 | 3.17E-13 | 173480 |
| rs1794279 | T | G | Granulocyte count | 27863252 | 2016 | -0.07112 | 0.005338 | 1.71E-40 | 173480 |
| rs1794279 | T | G | Hematocrit | 27863252 | 2016 | -0.04609 | 0.005249 | 1.64E-18 | 173480 |
| rs1794279 | T | G | Hemoglobin concentration | 27863252 | 2016 | -0.05185 | 0.005273 | 8.14E-23 | 173480 |
| rs1794279 | T | G | High light scatter percentage of red cells | 27863252 | 2016 | -0.03655 | 0.005331 | 7.09E-12 | 173480 |
| rs1794279 | T | G | High light scatter reticulocyte count | 27863252 | 2016 | -0.04481 | 0.005331 | 4.28E-17 | 173480 |
| rs1794279 | T | G | Lymphocyte count | 27863252 | 2016 | -0.0821 | 0.005351 | 3.96E-53 | 173480 |
| rs1794279 | T | G | Monocyte count | 27863252 | 2016 | -0.07336 | 0.00531 | 2.02E-43 | 173480 |
| rs1794279 | T | G | Myeloid white cell count | 27863252 | 2016 | -0.07639 | 0.005352 | 3.20E-46 | 173480 |
| rs1794279 | T | G | Neutrophil count | 27863252 | 2016 | -0.06422 | 0.005323 | 1.64E-33 | 173480 |
| rs1794279 | T | G | Neutrophil percentage of granulocytes | 27863252 | 2016 | 0.037 | 0.005321 | 3.58E-12 | 173480 |
| rs1794279 | T | G | Platelet count | 27863252 | 2016 | -0.03566 | 0.005423 | 4.86E-11 | 173480 |
| rs1794279 | T | G | Plateletcrit | 27863252 | 2016 | -0.03023 | 0.005436 | 2.68E-08 | 173480 |
| rs1794279 | T | G | Red blood cell count | 27863252 | 2016 | -0.05265 | 0.005281 | 2.07E-23 | 173480 |
| rs1794279 | T | G | Red cell distribution width | 27863252 | 2016 | 0.02825 | 0.005266 | 8.13E-08 | 173480 |
| rs1794279 | T | G | Reticulocyte count | 27863252 | 2016 | -0.069 | 0.005341 | 3.48E-38 | 173480 |
| rs1794279 | T | G | Reticulocyte fraction of red cells | 27863252 | 2016 | -0.05748 | 0.005337 | 4.79E-27 | 173480 |
| rs1794279 | T | G | Sum basophil neutrophil counts | 27863252 | 2016 | -0.06522 | 0.005333 | 2.14E-34 | 173480 |
| rs1794279 | T | G | Sum eosinophil basophil counts | 27863252 | 2016 | -0.07393 | 0.00531 | 4.61E-44 | 173480 |
| rs1794279 | T | G | Sum neutrophil eosinophil counts | 27863252 | 2016 | -0.07016 | 0.005329 | 1.38E-39 | 173480 |
| rs1794279 | T | G | White blood cell count | 27863252 | 2016 | -0.09623 | 0.005328 | 6.38E-73 | 173480 |
| rs1794279 | T | G | IgA deficiency | 27723758 | 2016 | 1.279 | 0.06114 | 3.10E-97 | 6487 |
| rs1794279 | T | G | Primary sclerosing cholangitis | 27992413 | 2017 | 1.092 | 0.044 | 5.23E-151 | 14890 |
| rs1794279 | T | G | Crohns disease progression | 28067912 | 2017 | -0.4162 | 0.09164 | 5.58E-06 | 2734 |
| rs1794279 | T | G | Arm fat mass left | UKBB | 2017 | 0.01681 | 0.003588 | 2.78E-06 | 331164 |
| rs1794279 | T | G | Arm fat mass right | UKBB | 2017 | 0.01663 | 0.003587 | 3.56E-06 | 331226 |
| rs1794279 | T | G | Arm fat-free mass left | UKBB | 2017 | 0.02307 | 0.002327 | 3.65E-23 | 331159 |
| rs1794279 | T | G | Arm fat-free mass right | UKBB | 2017 | 0.02282 | 0.002281 | 1.44E-23 | 331221 |
| rs1794279 | T | G | Arm predicted mass left | UKBB | 2017 | 0.02294 | 0.002318 | 4.36E-23 | 331146 |
| rs1794279 | T | G | Arm predicted mass right | UKBB | 2017 | 0.02244 | 0.002273 | 5.44E-23 | 331216 |
| rs1794279 | T | G | Asthma | UKBB | 2017 | 0.01021 | 0.001173 | 3.07E-18 | 336782 |
| rs1794279 | T | G | Basal metabolic rate | UKBB | 2017 | 0.02607 | 0.002427 | 6.65E-27 | 331307 |
| rs1794279 | T | G | Comparative body size at age 10 | UKBB | 2017 | 0.0182 | 0.002507 | 3.90E-13 | 331693 |
| rs1794279 | T | G | Comparative height size at age 10 | UKBB | 2017 | 0.01732 | 0.002499 | 4.20E-12 | 332021 |
| rs1794279 | T | G | Diabetes diagnosed by doctor | UKBB | 2017 | 0.004903 | 0.0007846 | 4.15E-10 | 336473 |
| rs1794279 | T | G | Diastolic blood pressure | UKBB | 2017 | -0.02875 | 0.003716 | 1.02E-14 | 317756 |
| rs1794279 | T | G | Doctor diagnosed sarcoidosis | UKBB | 2017 | 0.004756 | 0.0004731 | 9.39E-24 | 83529 |
| rs1794279 | T | G | Eye problems or disorders: diabetes related eye disease | UKBB | 2017 | 0.004643 | 0.0008853 | 1.57E-07 | 108817 |
| rs1794279 | T | G | Forced expiratory volume in 1-second, predicted percentage | UKBB | 2017 | -0.04096 | 0.006362 | 1.21E-10 | 110423 |
| rs1794279 | T | G | Height | UKBB | 2017 | 0.03095 | 0.0026 | 1.17E-32 | 336474 |
| rs1794279 | T | G | Hip circumference | UKBB | 2017 | 0.0264 | 0.003625 | 3.27E-13 | 336601 |
| rs1794279 | T | G | Hordeolum and chalazion | UKBB | 2017 | -0.000944 | 0.0002114 | 8.00E-06 | 337199 |
| rs1794279 | T | G | Illnesses of father: lung cancer | UKBB | 2017 | 0.00618 | 0.00111 | 2.61E-08 | 294142 |
| rs1794279 | T | G | Illnesses of siblings: diabetes | UKBB | 2017 | 0.005964 | 0.001128 | 1.24E-07 | 261075 |
| rs1794279 | T | G | Impedance of arm left | UKBB | 2017 | -0.01146 | 0.002585 | 9.33E-06 | 331292 |
| rs1794279 | T | G | Impedance of arm right | UKBB | 2017 | -0.01157 | 0.002574 | 6.94E-06 | 331279 |
| rs1794279 | T | G | Impedance of leg left | UKBB | 2017 | -0.02475 | 0.003337 | 1.22E-13 | 331296 |
| rs1794279 | T | G | Impedance of leg right | UKBB | 2017 | -0.02361 | 0.003312 | 1.01E-12 | 331301 |
| rs1794279 | T | G | Impedance of whole body | UKBB | 2017 | -0.01721 | 0.002791 | 7.02E-10 | 331284 |
| rs1794279 | T | G | Insulin-dependent diabetes mellitus | UKBB | 2017 | 0.000884 | 0.0001312 | 1.60E-11 | 337199 |
| rs1794279 | T | G | Intestinal malabsorption | UKBB | 2017 | 0.004768 | 0.0001576 | 9.66E-201 | 337199 |
| rs1794279 | T | G | Leg fat-free mass left | UKBB | 2017 | 0.02593 | 0.00241 | 5.48E-27 | 331258 |
| rs1794279 | T | G | Leg fat-free mass right | UKBB | 2017 | 0.02656 | 0.002409 | 2.95E-28 | 331285 |
| rs1794279 | T | G | Leg predicted mass left | UKBB | 2017 | 0.02583 | 0.002394 | 3.88E-27 | 331253 |
| rs1794279 | T | G | Leg predicted mass right | UKBB | 2017 | 0.02627 | 0.002393 | 4.95E-28 | 331285 |
| rs1794279 | T | G | Long-standing illness, disability or infirmity | UKBB | 2017 | 0.01194 | 0.001736 | 6.09E-12 | 329663 |
| rs1794279 | T | G | Medication for cholesterol, blood pressure or diabetes: insulin | UKBB | 2017 | 0.006338 | 0.0006285 | 6.57E-24 | 154702 |
| rs1794279 | T | G | Medication for pain relief, constipation, heartburn: none of the above | UKBB | 2017 | 0.01105 | 0.001833 | 1.63E-09 | 333581 |
| rs1794279 | T | G | Medication for pain relief, constipation, heartburn: paracetamol | UKBB | 2017 | -0.009231 | 0.001516 | 1.12E-09 | 333581 |
| rs1794279 | T | G | Mouth or teeth dental problems: dentures | UKBB | 2017 | 0.008975 | 0.001376 | 6.94E-11 | 336138 |
| rs1794279 | T | G | Nervous feelings | UKBB | 2017 | -0.007682 | 0.001573 | 1.04E-06 | 328725 |
| rs1794279 | T | G | Number of days or week of moderate physical activity 10+ minutes | UKBB | 2017 | -0.04171 | 0.008759 | 1.92E-06 | 321309 |
| rs1794279 | T | G | Other anaemias | UKBB | 2017 | 0.001413 | 0.0003066 | 4.07E-06 | 337199 |
| rs1794279 | T | G | Other chronic obstructive pulmonary disease | UKBB | 2017 | 0.000838 | 0.0001867 | 7.08E-06 | 337199 |
| rs1794279 | T | G | Other predominantly sexually transmitted diseases | UKBB | 2017 | 0.000289 | 6.45E-05 | 7.10E-06 | 337199 |
| rs1794279 | T | G | Other serious medical condition or disability diagnosed by doctor | UKBB | 2017 | 0.009833 | 0.001497 | 5.03E-11 | 331274 |
| rs1794279 | T | G | Potassium in urine | UKBB | 2017 | -0.02134 | 0.003677 | 6.54E-09 | 326816 |
| rs1794279 | T | G | Seen a psychiatrist for nerves, anxiety, tension or depression | UKBB | 2017 | -0.006087 | 0.001165 | 1.74E-07 | 335888 |
| rs1794279 | T | G | Self-reported adrenocortical insufficiency or addisons disease | UKBB | 2017 | 0.000642 | 7.51E-05 | 1.20E-17 | 337159 |
| rs1794279 | T | G | Self-reported asthma | UKBB | 2017 | 0.01013 | 0.001175 | 6.72E-18 | 337159 |
| rs1794279 | T | G | Self-reported diabetes | UKBB | 2017 | 0.003764 | 0.0007116 | 1.22E-07 | 337159 |
| rs1794279 | T | G | Self-reported graves disease | UKBB | 2017 | 0.000301 | 5.18E-05 | 6.07E-09 | 337159 |
| rs1794279 | T | G | Self-reported hyperthyroidism or thyrotoxicosis | UKBB | 2017 | 0.007117 | 0.0003174 | 3.04E-111 | 337159 |
| rs1794279 | T | G | Self-reported hypothyroidism or myxoedema | UKBB | 2017 | 0.008206 | 0.0007816 | 8.79E-26 | 337159 |
| rs1794279 | T | G | Self-reported malabsorption or coeliac disease | UKBB | 2017 | 0.01271 | 0.0002395 | 0 | 337159 |
| rs1794279 | T | G | Self-reported psoriasis | UKBB | 2017 | -0.003212 | 0.0003912 | 2.22E-16 | 337159 |
| rs1794279 | T | G | Self-reported sarcoidosis | UKBB | 2017 | 0.001504 | 0.0001611 | 9.89E-21 | 337159 |
| rs1794279 | T | G | Self-reported sjogrens syndrome or sicca syndrome | UKBB | 2017 | 0.000714 | 0.0001097 | 7.12E-11 | 337159 |
| rs1794279 | T | G | Self-reported systemic lupus erythematosis or sle | UKBB | 2017 | 0.000905 | 0.0001209 | 7.02E-14 | 337159 |
| rs1794279 | T | G | Self-reported thyroid radioablation therapy | UKBB | 2017 | 0.000307 | 5.66E-05 | 5.58E-08 | 337159 |
| rs1794279 | T | G | Self-reported type 1 diabetes | UKBB | 2017 | 0.000807 | 0.0001069 | 4.30E-14 | 337159 |
| rs1794279 | T | G | Sitting height | UKBB | 2017 | 0.0416 | 0.002829 | 5.91E-49 | 336172 |
| rs1794279 | T | G | Started insulin within one year diagnosis of diabetes | UKBB | 2017 | 0.08096 | 0.005356 | 2.97E-51 | 15397 |
| rs1794279 | T | G | Thyrotoxicosis | UKBB | 2017 | 0.000715 | 0.0001093 | 5.92E-11 | 337199 |
| rs1794279 | T | G | Treatment with carbimazole | UKBB | 2017 | 0.000791 | 0.0001048 | 4.51E-14 | 337159 |
| rs1794279 | T | G | Treatment with ferrous sulphate | UKBB | 2017 | 0.001328 | 0.0002547 | 1.85E-07 | 337159 |
| rs1794279 | T | G | Treatment with fludrocortisone | UKBB | 2017 | 0.000635 | 7.69E-05 | 1.49E-16 | 337159 |
| rs1794279 | T | G | Treatment with insulin | UKBB | 2017 | 0.004757 | 0.0004381 | 1.89E-27 | 180203 |
| rs1794279 | T | G | Treatment with insulin product | UKBB | 2017 | 0.005423 | 0.0003624 | 1.30E-50 | 337159 |
| rs1794279 | T | G | Treatment with levothyroxine sodium | UKBB | 2017 | 0.008046 | 0.0007202 | 5.72E-29 | 337159 |
| rs1794279 | T | G | Treatment with paracetamol | UKBB | 2017 | -0.007368 | 0.001415 | 1.92E-07 | 337159 |
| rs1794279 | T | G | Treatment with thyroxine product | UKBB | 2017 | 0.002689 | 0.0003925 | 7.32E-12 | 337159 |
| rs1794279 | T | G | Treatment with ventolin 100micrograms inhaler | UKBB | 2017 | 0.003739 | 0.0006143 | 1.15E-09 | 337159 |
| rs1794279 | T | G | Trunk fat mass | UKBB | 2017 | 0.01751 | 0.003686 | 2.02E-06 | 331093 |
| rs1794279 | T | G | Trunk fat-free mass | UKBB | 2017 | 0.02556 | 0.002305 | 1.47E-28 | 331030 |
| rs1794279 | T | G | Trunk predicted mass | UKBB | 2017 | 0.02522 | 0.002298 | 5.20E-28 | 330995 |
| rs1794279 | T | G | Unspecified haematuria | UKBB | 2017 | -0.003535 | 0.0005364 | 4.39E-11 | 337199 |
| rs1794279 | T | G | Weight | UKBB | 2017 | 0.02791 | 0.003198 | 2.62E-18 | 336227 |
| rs1794279 | T | G | Whole body fat-free mass | UKBB | 2017 | 0.02615 | 0.002314 | 1.34E-29 | 331291 |
| rs1794279 | T | G | Whole body water mass | UKBB | 2017 | 0.02614 | 0.002318 | 1.73E-29 | 331315 |
| rs1794279 | T | G | Rheumatoid arthritis | 24390342 | 2014 | -0.3285 | 0.02837 | 9.00E-32 | 58284 |
| rs1794279 | T | G | Rheumatoid arthritis | 24390342 | 2014 | -0.3285 | 0.02837 | 8.30E-32 | 80799 |
| rs1794279 | T | G | Schizophrenia | 25056061 | 2014 | -0.1675 | 0.0192 | 3.07E-18 | 82315 |
| rs2160215 | C | T | Self-reported hyperthyroidism or thyrotoxicosis | UKBB | 2017 | 0.002516 | 0.0002181 | 8.90E-31 | 337159 |
| rs2160215 | C | T | Thyrotoxicosis | UKBB | 2017 | 0.000370 | 7.51E-05 | 8.17E-07 | 337199 |
| rs2160215 | C | T | Treatment with carbimazole | UKBB | 2017 | 0.000464 | 7.20E-05 | 1.10E-10 | 337159 |
| rs28360997 | A | G | Granulocyte count | 27863252 | 2016 | 0.04238 | 0.005004 | 2.45E-17 | 173480 |
| rs28360997 | A | G | High light scatter percentage of red cells | 27863252 | 2016 | -0.02626 | 0.004996 | 1.48E-07 | 173480 |
| rs28360997 | A | G | High light scatter reticulocyte count | 27863252 | 2016 | -0.02951 | 0.004996 | 3.52E-09 | 173480 |
| rs28360997 | A | G | Lymphocyte count | 27863252 | 2016 | 0.03893 | 0.005015 | 8.30E-15 | 173480 |
| rs28360997 | A | G | Mean corpuscular hemoglobin concentration | 27863252 | 2016 | -0.02326 | 0.004827 | 1.44E-06 | 173480 |
| rs28360997 | A | G | Mean platelet volume | 27863252 | 2016 | 0.04483 | 0.005052 | 7.03E-19 | 173480 |
| rs28360997 | A | G | Monocyte count | 27863252 | 2016 | 0.02899 | 0.004976 | 5.69E-09 | 173480 |
| rs28360997 | A | G | Myeloid white cell count | 27863252 | 2016 | 0.04393 | 0.005016 | 1.99E-18 | 173480 |
| rs28360997 | A | G | Neutrophil count | 27863252 | 2016 | 0.04247 | 0.004989 | 1.70E-17 | 173480 |
| rs28360997 | A | G | Reticulocyte count | 27863252 | 2016 | -0.02805 | 0.005006 | 2.10E-08 | 173480 |
| rs28360997 | A | G | Reticulocyte fraction of red cells | 27863252 | 2016 | -0.0238 | 0.005002 | 1.95E-06 | 173480 |
| rs28360997 | A | G | Sum basophil neutrophil counts | 27863252 | 2016 | 0.0427 | 0.004998 | 1.32E-17 | 173480 |
| rs28360997 | A | G | Sum neutrophil eosinophil counts | 27863252 | 2016 | 0.04214 | 0.004994 | 3.21E-17 | 173480 |
| rs28360997 | A | G | White blood cell count | 27863252 | 2016 | 0.05297 | 0.004992 | 2.67E-26 | 173480 |
| rs28360997 | A | G | Primary sclerosing cholangitis | 27992413 | 2017 | -0.1914 | 0.042 | 2.93E-06 | 14890 |
| rs28360997 | A | G | Intestinal malabsorption | UKBB | 2017 | -0.001085 | 0.000147 | 1.59E-13 | 337199 |
| rs28360997 | A | G | Peak expiratory flow | UKBB | 2017 | 0.01544 | 0.003032 | 3.54E-07 | 307638 |
| rs28360997 | A | G | Self-reported hyperthyroidism or thyrotoxicosis | UKBB | 2017 | -0.00172 | 0.0002958 | 6.01E-09 | 337159 |
| rs28360997 | A | G | Self-reported hypothyroidism or myxoedema | UKBB | 2017 | 0.007081 | 0.0007277 | 2.25E-22 | 337159 |
| rs28360997 | A | G | Self-reported malabsorption or coeliac disease | UKBB | 2017 | -0.002462 | 0.0002239 | 4.00E-28 | 337159 |
| rs28360997 | A | G | Treatment with levothyroxine sodium | UKBB | 2017 | 0.005286 | 0.0006707 | 3.22E-15 | 337159 |
| rs28360997 | A | G | Rheumatoid arthritis | 24390342 | 2014 | -0.1863 | 0.04313 | 5.00E-06 | 22515 |
| rs28360997 | A | G | Rheumatoid arthritis | 24390342 | 2014 | 0.1222 | 0.02024 | 2.00E-09 | 58284 |
| rs28360997 | A | G | Schizophrenia | 25056061 | 2014 | 0.0724 | 0.0159 | 5.54E-06 | 82315 |
| rs28383364 | A | G | Hematocrit | 27863252 | 2016 | -0.02212 | 0.004414 | 5.44E-07 | 173480 |
| rs28383364 | A | G | Hemoglobin concentration | 27863252 | 2016 | -0.02496 | 0.004434 | 1.81E-08 | 173480 |
| rs28383364 | A | G | Lymphocyte count | 27863252 | 2016 | -0.02837 | 0.004502 | 2.94E-10 | 173480 |
| rs28383364 | A | G | Reticulocyte count | 27863252 | 2016 | -0.02038 | 0.004491 | 5.65E-06 | 173480 |
| rs28383364 | A | G | White blood cell count | 27863252 | 2016 | -0.02272 | 0.004483 | 4.02E-07 | 173480 |
| rs28383364 | A | G | IgA deficiency | 27723758 | 2016 | -0.4545 | 0.05943 | 2.05E-14 | 6487 |
| rs28383364 | A | G | Asthma | UKBB | 2017 | 0.004485 | 0.0009818 | 4.93E-06 | 336782 |
| rs28383364 | A | G | Insulin-dependent diabetes mellitus | UKBB | 2017 | 0.000721 | 0.0001099 | 5.19E-11 | 337199 |
| rs28383364 | A | G | Long-standing illness, disability or infirmity | UKBB | 2017 | 0.01002 | 0.001454 | 5.42E-12 | 329663 |
| rs28383364 | A | G | Medication for cholesterol, blood pressure or diabetes: insulin | UKBB | 2017 | 0.003818 | 0.000528 | 4.87E-13 | 154702 |
| rs28383364 | A | G | Nasal polyp | UKBB | 2017 | 0.001246 | 0.0002136 | 5.53E-09 | 337199 |
| rs28383364 | A | G | No blood clot, bronchitis, emphysema, asthma, rhinitis, eczema or allergy diagnosed by doctor | UKBB | 2017 | -0.006629 | 0.001436 | 3.88E-06 | 336782 |
| rs28383364 | A | G | Number of treatments or medications taken | UKBB | 2017 | 0.0115 | 0.002458 | 2.88E-06 | 337159 |
| rs28383364 | A | G | Other eye problems | UKBB | 2017 | 0.004851 | 0.001075 | 6.43E-06 | 336428 |
| rs28383364 | A | G | Other rheumatoid arthritis | UKBB | 2017 | 0.001348 | 0.0001641 | 2.16E-16 | 337199 |
| rs28383364 | A | G | Other serious medical condition or disability diagnosed by doctor | UKBB | 2017 | 0.007292 | 0.001253 | 5.86E-09 | 331274 |
| rs28383364 | A | G | Psoriasis | UKBB | 2017 | -0.000463 | 9.79E-05 | 2.21E-06 | 337199 |
| rs28383364 | A | G | Self-reported ankylosing spondylitis | UKBB | 2017 | 0.000731 | 0.0001645 | 8.70E-06 | 337159 |
| rs28383364 | A | G | Self-reported asthma | UKBB | 2017 | 0.004698 | 0.0009836 | 1.78E-06 | 337159 |
| rs28383364 | A | G | Self-reported bronchiectasis | UKBB | 2017 | 0.000733 | 0.0001517 | 1.35E-06 | 337159 |
| rs28383364 | A | G | Self-reported eczema or dermatitis | UKBB | 2017 | 0.003271 | 0.000488 | 2.05E-11 | 337159 |
| rs28383364 | A | G | Self-reported hyperthyroidism or thyrotoxicosis | UKBB | 2017 | 0.003302 | 0.0002659 | 2.11E-35 | 337159 |
| rs28383364 | A | G | Self-reported hypothyroidism or myxoedema | UKBB | 2017 | 0.006826 | 0.0006544 | 1.81E-25 | 337159 |
| rs28383364 | A | G | Self-reported multiple sclerosis | UKBB | 2017 | 0.00129 | 0.0001852 | 3.30E-12 | 337159 |
| rs28383364 | A | G | Self-reported psoriasis | UKBB | 2017 | -0.006922 | 0.0003273 | 3.55E-99 | 337159 |
| rs28383364 | A | G | Self-reported psoriatic arthropathy | UKBB | 2017 | -0.000751 | 0.0001349 | 2.58E-08 | 337159 |
| rs28383364 | A | G | Self-reported rheumatoid arthritis | UKBB | 2017 | 0.003707 | 0.0003214 | 9.23E-31 | 337159 |
| rs28383364 | A | G | Self-reported type 1 diabetes | UKBB | 2017 | 0.000631 | 8.95E-05 | 1.78E-12 | 337159 |
| rs28383364 | A | G | Seropositive rheumatoid arthritis | UKBB | 2017 | 0.000398 | 7.51E-05 | 1.11E-07 | 337199 |
| rs28383364 | A | G | Started insulin within one year diagnosis of diabetes | UKBB | 2017 | 0.05235 | 0.004677 | 5.76E-29 | 15397 |
| rs28383364 | A | G | Taking other prescription medications | UKBB | 2017 | 0.008192 | 0.001535 | 9.53E-08 | 336330 |
| rs28383364 | A | G | Thyrotoxicosis | UKBB | 2017 | 0.000464 | 9.15E-05 | 3.88E-07 | 337199 |
| rs28383364 | A | G | Treatment with calcipotriol | UKBB | 2017 | -0.000439 | 7.41E-05 | 3.05E-09 | 337159 |
| rs28383364 | A | G | Treatment with dovobet ointment | UKBB | 2017 | -0.000740 | 0.0001042 | 1.23E-12 | 337159 |
| rs28383364 | A | G | Treatment with dovonex 50micrograms or g cream | UKBB | 2017 | -0.000481 | 7.43E-05 | 9.03E-11 | 337159 |
| rs28383364 | A | G | Treatment with insulin | UKBB | 2017 | 0.002537 | 0.0003658 | 4.07E-12 | 180203 |
| rs28383364 | A | G | Treatment with insulin product | UKBB | 2017 | 0.003061 | 0.0003035 | 6.35E-24 | 337159 |
| rs28383364 | A | G | Treatment with levothyroxine sodium | UKBB | 2017 | 0.006333 | 0.000603 | 8.46E-26 | 337159 |
| rs28383364 | A | G | Treatment with methotrexate | UKBB | 2017 | 0.001335 | 0.0002227 | 2.05E-09 | 337159 |
| rs28383364 | A | G | Treatment with prednisolone | UKBB | 2017 | 0.001153 | 0.0002326 | 7.17E-07 | 337159 |
| rs28383364 | A | G | Treatment with sulfasalazine | UKBB | 2017 | 0.000615 | 0.0001347 | 4.83E-06 | 337159 |
| rs28383364 | A | G | Treatment with thyroxine product | UKBB | 2017 | 0.001735 | 0.0003286 | 1.30E-07 | 337159 |
| rs28383364 | A | G | IgG digalactosylation | 28878392 | 2017 | NA | NA | 2.38E-06 | 1960 |
| rs28383364 | A | G | IgG galactosylation | 28878392 | 2017 | NA | NA | 1.34E-08 | 1960 |
| rs28383364 | A | G | IgG N-glycosylation | 28878392 | 2017 | NA | NA | 2.05E-07 | 1960 |
| rs3087243 | A | G | Alopecia areata | 20596022 | 2010 | NA | NA | 6.00E-12 | 4332 |
| rs3087243 | A | G | Graves disease | 21829393 | 2011 | NA | NA | 1.00E-21 | 12501 |
| rs3087243 | A | G | Rheumatoid arthritis | 20453842 | 2010 | NA | NA | 1.20E-08 | 25708 |
| rs3087243 | A | G | Rheumatoid arthritis | 21383967 | 2011 | NA | NA | 1.64E-08 | 38053 |
| rs3087243 | A | G | Rheumatoid arthritis | 23143596 | 2012 | NA | NA | 1.42E-11 | 27345 |
| rs3087243 | A | G | Rheumatoid arthritis cyclic citrullinated peptide CCP positive | 23143596 | 2012 | NA | NA | 3.88E-11 | 27345 |
| rs3087243 | A | G | Selective immunoglobulin A deficiency IgAD | 20694011 | 2010 | NA | NA | 1.20E-15 | 1520 |
| rs3087243 | A | G | Type 1 diabetes | 17554300 | 2007 | NA | NA | 2.07E-07 | 4806 |
| rs3087243 | A | G | Type 1 diabetes | 18978792 | 2008 | NA | NA | 7.67E-11 | 8207 |
| rs3087243 | A | G | Type 1 diabetes | 19430480 | 2009 | NA | NA | 1.20E-15 | 16559 |
| rs3087243 | A | G | Type 1 diabetes | 21829393 | 2011 | NA | NA | 2.30E-17 | 12501 |
| rs3087243 | A | G | Type 1 diabetes | 21980299 | 2011 | NA | NA | 1.42E-13 | 26890 |
| rs3087243 | A | G | Hypothyroidism | 27182965 | 2016 | NA | NA | 1.00E-15 | NA |
| rs3087243 | A | G | Rheumatoid arthritis | 20453842 | 2010 | -0.1398 | 0.02439 | 1.00E-08 | NA |
| rs3087243 | A | G | Rheumatoid arthritis | 24390342 | 2014 | NA | NA | 4.00E-22 | NA |
| rs3087243 | A | G | Rheumatoid arthritis | 24390342 | 2014 | NA | NA | 3.00E-25 | NA |
| rs3087243 | A | G | Type 1 diabetes | 18978792 | 2008 | NA | NA | 8.00E-11 | NA |
| rs3087243 | A | G | Type 1 diabetes | 19430480 | 2009 | NA | NA | 1.00E-15 | NA |
| rs3087243 | A | G | Type 1 diabetes autoantibodies | 21829393 | 2011 | NA | NA | 2.00E-17 | NA |
| rs3087243 | A | G | Long-standing illness, disability or infirmity | UKBB | 2017 | -0.00529 | 0.001157 | 4.87E-06 | 329663 |
| rs3087243 | A | G | Number of self-reported non-cancer illnesses | UKBB | 2017 | -0.01003 | 0.002016 | 6.49E-07 | 337159 |
| rs3087243 | A | G | Other serious medical condition or disability diagnosed by doctor | UKBB | 2017 | -0.004827 | 0.0009975 | 1.31E-06 | 331274 |
| rs3087243 | A | G | Self-reported hyperthyroidism or thyrotoxicosis | UKBB | 2017 | -0.001999 | 0.0002117 | 3.80E-21 | 337159 |
| rs3087243 | A | G | Self-reported hypothyroidism or myxoedema | UKBB | 2017 | -0.008497 | 0.0005209 | 8.63E-60 | 337159 |
| rs3087243 | A | G | Started insulin within one year diagnosis of diabetes | UKBB | 2017 | -0.01688 | 0.003718 | 5.63E-06 | 15397 |
| rs3087243 | A | G | Taking other prescription medications | UKBB | 2017 | -0.006075 | 0.001222 | 6.69E-07 | 336330 |
| rs3087243 | A | G | Treatment with levothyroxine sodium | UKBB | 2017 | -0.007349 | 0.0004801 | 7.08E-53 | 337159 |
| rs3087243 | A | G | Treatment with prednisolone | UKBB | 2017 | -0.000837 | 0.0001852 | 6.13E-06 | 337159 |
| rs3087243 | A | G | Treatment with thyroxine product | UKBB | 2017 | -0.001857 | 0.0002616 | 1.27E-12 | 337159 |
| rs3087243 | A | G | Rheumatoid arthritis | 24390342 | 2014 | -0.1393 | 0.0176 | 9.20E-20 | 58284 |
| rs3087243 | A | G | Rheumatoid arthritis | 24390342 | 2014 | -0.1393 | 0.01458 | 1.70E-22 | 80799 |
| rs3087243 | A | G | Rheumatoid arthritis | 20453842 | 2010 | -0.1393 | 0.02347 | 2.24E-08 | 25695 |
| rs3087243 | A | G | Arthritis rheumatoid | 20453842 | 2010 | NA | NA | 1.00E-08 | NA |
| rs3087243 | A | G | Diabetes mellitus type 1 | 18978792 | 2008 | NA | NA | 8.00E-11 | NA |
| rs3087243 | A | G | Diabetes mellitus type 1 | 19430480 | 2009 | NA | NA | 1.00E-15 | NA |
| rs3087243 | A | G | Diabetes mellitus type 1 | 21829393 | 2011 | NA | NA | 2.00E-17 | NA |
| rs6679677 | A | C | Basophil percentage of granulocytes | 27863252 | 2016 | 0.0306 | 0.005773 | 1.16E-07 | 173480 |
| rs6679677 | A | C | Basophil percentage of white cells | 27863252 | 2016 | 0.03124 | 0.005722 | 4.77E-08 | 173480 |
| rs6679677 | A | C | Granulocyte count | 27863252 | 2016 | -0.04239 | 0.005918 | 7.87E-13 | 173480 |
| rs6679677 | A | C | Lymphocyte count | 27863252 | 2016 | -0.05556 | 0.005932 | 7.57E-21 | 173480 |
| rs6679677 | A | C | Myeloid white cell count | 27863252 | 2016 | -0.04242 | 0.005932 | 8.59E-13 | 173480 |
| rs6679677 | A | C | Neutrophil count | 27863252 | 2016 | -0.04258 | 0.005901 | 5.35E-13 | 173480 |
| rs6679677 | A | C | Sum basophil neutrophil counts | 27863252 | 2016 | -0.04266 | 0.005911 | 5.31E-13 | 173480 |
| rs6679677 | A | C | Sum neutrophil eosinophil counts | 27863252 | 2016 | -0.04246 | 0.005909 | 6.68E-13 | 173480 |
| rs6679677 | A | C | White blood cell count | 27863252 | 2016 | -0.05549 | 0.005907 | 5.72E-21 | 173480 |
| rs6679677 | A | C | Amoxicillin clavulanate drug induced liver injury | 21570397 | 2011 | NA | NA | 8.70E-06 | 733 |
| rs6679677 | A | C | Crohns disease | 18587394 | 2008 | NA | NA | 4.95E-09 | 8059 |
| rs6679677 | A | C | Crohns disease | 23128233 | 2012 | NA | NA | 2.03E-15 | 34366 |
| rs6679677 | A | C | Drug induced liver injury all cholestatic DILI cases | 22968431 | 2012 | NA | NA | 1.35E-06 | 3784 |
| rs6679677 | A | C | Hypothyroidism | 22493691 | 2012 | NA | NA | 2.80E-13 | 39282 |
| rs6679677 | A | C | Juvenile idiopathic arthritis | 22354554 | 2012 | NA | NA | 1.07E-06 | 3872 |
| rs6679677 | A | C | Juvenile idiopathic arthritis including oligoarticular and rheumatoid factor negative polyarticular JIA | 23603761 | 2013 | NA | NA | 3.19E-25 | 15872 |
| rs6679677 | A | C | Oligoarticular juvenile idiopathic arthritis | 23603761 | 2013 | NA | NA | 1.11E-18 | 15872 |
| rs6679677 | A | C | Rheumatoid arthritis | 18794853 | 2008 | NA | NA | 5.70E-42 | 15853 |
| rs6679677 | A | C | Rheumatoid arthritis | 20453842 | 2010 | NA | NA | 4.39E-70 | 25708 |
| rs6679677 | A | C | Rheumatoid factor negative polyarticular juvenile idiopathic arthritis | 23603761 | 2013 | NA | NA | 3.45E-11 | 15872 |
| rs6679677 | A | C | Selective immunoglobulin A deficiency IgAD | 20694011 | 2010 | NA | NA | 6.00E-42 | 1520 |
| rs6679677 | A | C | Type 1 diabetes | 17554300 | 2007 | NA | NA | 1.40E-41 | 4806 |
| rs6679677 | A | C | Type 1 diabetes | 18978792 | 2008 | NA | NA | 1.27E-40 | 8207 |
| rs6679677 | A | C | Crohns disease | 23128233 | 2012 | NA | NA | 5.00E-09 | 14342 |
| rs6679677 | A | C | Crohns disease | 26192919 | 2015 | -0.2172 | 0.0429 | 4.17E-07 | 20883 |
| rs6679677 | A | C | Antineutrophil cytoplasmic antibody associated vasculitis | 28029757 | 2016 | NA | NA | 2.00E-08 | NA |
| rs6679677 | A | C | Crohns disease | 23128233 | 2012 | NA | NA | 2.00E-15 | NA |
| rs6679677 | A | C | Hypothyroidism | 22493691 | 2012 | 0.3082 | 0.04225 | 3.00E-13 | NA |
| rs6679677 | A | C | Monokine induced by gamma interferon levels | 27989323 | 2017 | -0.162 | 0.03298 | 9.00E-07 | NA |
| rs6679677 | A | C | Pediatric autoimmune diseases | 26301688 | 2015 | NA | NA | 8.00E-11 | NA |
| rs6679677 | A | C | Rheumatoid arthritis | 17554300 | 2007 | 0.6831 | 0.06622 | 6.00E-25 | NA |
| rs6679677 | A | C | Rheumatoid arthritis | 18794853 | 2008 | NA | NA | 6.00E-42 | NA |
| rs6679677 | A | C | Systemic lupus erythematosus | 26502338 | 2015 | 0.3293 | 0.04103 | 1.00E-15 | NA |
| rs6679677 | A | C | Type 1 diabetes | 17554260 | 2007 | 0.6366 | 0.06326 | 8.00E-24 | NA |
| rs6679677 | A | C | Type 1 diabetes | 17554300 | 2007 | 0.5988 | 0.05675 | 5.00E-26 | NA |
| rs6679677 | A | C | Type 1 diabetes | 18978792 | 2008 | NA | NA | 1.00E-40 | NA |
| rs6679677 | A | C | Diabetes diagnosed by doctor | UKBB | 2017 | 0.004567 | 0.000857 | 9.88E-08 | 336473 |
| rs6679677 | A | C | Insulin-dependent diabetes mellitus | UKBB | 2017 | 0.000813 | 0.0001433 | 1.37E-08 | 337199 |
| rs6679677 | A | C | Long-standing illness, disability or infirmity | UKBB | 2017 | 0.01442 | 0.001896 | 2.88E-14 | 329663 |
| rs6679677 | A | C | Medication for cholesterol, blood pressure or diabetes: insulin | UKBB | 2017 | 0.005448 | 0.000686 | 2.00E-15 | 154702 |
| rs6679677 | A | C | Number of self-reported non-cancer illnesses | UKBB | 2017 | 0.01682 | 0.003302 | 3.49E-07 | 337159 |
| rs6679677 | A | C | Number of treatments or medications taken | UKBB | 2017 | 0.01842 | 0.003206 | 9.17E-09 | 337159 |
| rs6679677 | A | C | Other rheumatoid arthritis | UKBB | 2017 | 0.001127 | 0.0002141 | 1.40E-07 | 337199 |
| rs6679677 | A | C | Other serious medical condition or disability diagnosed by doctor | UKBB | 2017 | 0.01176 | 0.001634 | 6.12E-13 | 331274 |
| rs6679677 | A | C | Self-reported diabetes | UKBB | 2017 | 0.003986 | 0.0007771 | 2.90E-07 | 337159 |
| rs6679677 | A | C | Self-reported hyperthyroidism or thyrotoxicosis | UKBB | 2017 | 0.002618 | 0.0003469 | 4.42E-14 | 337159 |
| rs6679677 | A | C | Self-reported hypothyroidism or myxoedema | UKBB | 2017 | 0.0201 | 0.000853 | 1.07E-122 | 337159 |
| rs6679677 | A | C | Self-reported pernicious anaemia | UKBB | 2017 | 0.001065 | 0.0002202 | 1.31E-06 | 337159 |
| rs6679677 | A | C | Self-reported rheumatoid arthritis | UKBB | 2017 | 0.002726 | 0.0004193 | 7.94E-11 | 337159 |
| rs6679677 | A | C | Self-reported type 1 diabetes | UKBB | 2017 | 0.000536 | 0.0001168 | 4.38E-06 | 337159 |
| rs6679677 | A | C | Started insulin within one year diagnosis of diabetes | UKBB | 2017 | 0.05595 | 0.005856 | 1.43E-21 | 15397 |
| rs6679677 | A | C | Taking other prescription medications | UKBB | 2017 | 0.01598 | 0.002002 | 1.44E-15 | 336330 |
| rs6679677 | A | C | Treatment with folic acid product | UKBB | 2017 | 0.001789 | 0.0003626 | 8.02E-07 | 337159 |
| rs6679677 | A | C | Treatment with insulin | UKBB | 2017 | 0.003193 | 0.0004787 | 2.57E-11 | 180203 |
| rs6679677 | A | C | Treatment with insulin product | UKBB | 2017 | 0.00387 | 0.0003958 | 1.42E-22 | 337159 |
| rs6679677 | A | C | Treatment with levothyroxine sodium | UKBB | 2017 | 0.01513 | 0.0007863 | 1.91E-82 | 337159 |
| rs6679677 | A | C | Treatment with methotrexate | UKBB | 2017 | 0.002405 | 0.0002905 | 1.25E-16 | 337159 |
| rs6679677 | A | C | Treatment with thyroxine product | UKBB | 2017 | 0.005396 | 0.0004285 | 2.40E-36 | 337159 |
| rs6679677 | A | C | Treatment with thyroxine sodium | UKBB | 2017 | 0.001188 | 0.0002112 | 1.85E-08 | 337159 |
| rs6679677 | A | C | Rheumatoid arthritis | 24390342 | 2014 | 0.5933 | 0.02256 | 3.10E-149 | 58284 |
| rs6679677 | A | C | Rheumatoid arthritis | 24390342 | 2014 | 0.5933 | 0.02256 | 2.10E-149 | 80799 |
| rs6679677 | A | C | Rheumatoid arthritis | 20453842 | 2010 | 0.6627 | 0.03811 | 4.39E-70 | 25689 |
| rs6679677 | A | C | Arthritis rheumatoid | 17554300 | 2007 | NA | NA | 6.00E-25 | NA |
| rs6679677 | A | C | Arthritis rheumatoid | 18794853 | 2008 | NA | NA | 6.00E-42 | NA |
| rs6679677 | A | C | Diabetes mellitus type 1 | 17554260 | 2007 | NA | NA | 8.00E-24 | NA |
| rs6679677 | A | C | Diabetes mellitus type 1 | 17554300 | 2007 | NA | NA | 5.00E-26 | NA |
| rs6679677 | A | C | Diabetes mellitus type 1 | 18978792 | 2008 | NA | NA | 1.00E-40 | NA |
| rs6679677 | A | C | Coronary artery disease | 29212778 | 2018 | 0.0437 | 0.0088 | 7.07E-07 | 547261 |

**Supplementary Material 3 SNPs associated with OA.**

| SNP | Chr | Position | Effect  allele | Other allele | EAF | Beta | Se | *P* for OA | *P* for hyperthyroidism |
| --- | --- | --- | --- | --- | --- | --- | --- | --- | --- |
| rs10405617 | 19 | 10752968 | G | A | 0.664222 | -0.00331891 | 0.0006 | 4.30E-08 | 0.628614 |
| rs12133235 | 1 | 150165849 | C | A | 0.42131 | 0.00331311 | 0.0005 | 1.20E-08 | 0.796656 |
| rs13107325 | 4 | 103188709 | T | C | 0.074913 | 0.00962712 | 0.0011 | 9.00E-19 | 0.208635 |
| rs2290573 | 15 | 75129594 | A | G | 0.559693 | 0.0038676 | 0.0006 | 2.90E-11 | 0.0155085 |
| rs3771501 | 2 | 70717653 | G | A | 0.524169 | -0.00425112 | 0.0006 | 1.40E-13 | 0.315766 |
| rs75621460 | 19 | 41833784 | A | G | 0.027098 | 0.0131574 | 0.0019 | 1.20E-11 | 0.946168 |

Chr: Chromosome; EAF: effect allele frequency; OA: osteoarthritis.
